# Effect of excise tax on sugar-sweetened beverages in Catalonia, Spain, three and a half years after its introduction

**DOI:** 10.1186/s12966-022-01262-8

**Published:** 2022-03-12

**Authors:** Miguel Ángel Royo-Bordonada, Carlos Fernández-Escobar, Carlos José Gil-Bellosta, Elena Ordaz

**Affiliations:** 1 National School of Public Health, Institute of Health Carlos III, Sinesio Delgado, 8, 28029 Madrid, Spain; 2 Circiter, S.L, Madrid, Spain

**Keywords:** Taxes, Sugar-sweetened beverages, Obesity prevention, Fiscal policy, Spain, Catalonia

## Abstract

**Background:**

The World Health Organisation urges countries to levy specific excise taxes on SSBs. Currently, more than 50 countries have introduced some type of tax on SSBs. In March 2017, the Autonomous Region of Catalonia approved the introduction of a tiered excise tax on SSBs for public health reasons. To evaluate the effect of the Catalonian excise tax on the price and purchase of sugar-sweetened beverages (SSBs) and their possible substitutes, i.e., non-sugar-sweetened beverages (NSSBs) and bottled water, three and half years after its introduction, and 1 year after the outbreak of the COVID-19 pandemic.

**Methods:**

We analysed purchase data on soft drinks, fruit drinks and water, sourced from the Ministry of Agriculture food-consumption panel, in a random sample of 12,500 households across Spain. We applied the synthetic control method to infer the causal impact of the intervention, based on a Bayesian structural time-series model which predicts the counterfactual response that would have occurred in Catalonia, had no intervention taken place.

**Results:**

As compared to the predicted (counterfactual) response, per capita purchases of SSBs fell by 0.17 l three and a half years after implementing the SSB tax in Catalonia, a 16.7% decline (95% CI: − 23.18, − 8.74). The mean SSB price rose by 0.11 €/L, an 11% increase (95% CI: 9.0, 14.1). Although there were no changes in mean NSSB prices, NSSB consumption rose by 0.19 l per capita, a 21.7% increase (95% CI: 18.25, 25.54). There were no variations in the price or consumption of bottled water. The effects were progressively greater over time, with SSB purchases decreasing by 10.4% at 1 year, 12.3% at 2 years, 15.3% at 3 years, and 16.7% at three and a half years of the tax’s introduction.

**Conclusions:**

The Catalonian SSB excise tax had a sustained and progressive impact over time, with a fall in consumption of as much as 16.7% three and half years after its introduction. The observed NSSB substitution effect should be borne in mind when considering the application of this type of tax to the rest of Spain.

**Supplementary Information:**

The online version contains supplementary material available at 10.1186/s12966-022-01262-8.

## Introduction

A quarter of all Spanish adults lives with general obesity and a third with abdominal obesity [1]. This health problem is also very common among the child and adolescent populations, with figures of around 10% for general obesity and 20% for abdominal obesity [2, 3]. It is especially worrying that over 100,000 primary school children, 2.5% of the population aged 6 to 9 years, may live with severe obesity in Spain, one of the countries most affected by this problem in Europe [4]. Persons with obesity have a lower life expectancy, which may be shortened by up to 10 years in cases of severe obesity, due to its contribution to the development of major non-communicable chronic diseases, such as cardiovascular disease and cancer [5].

Consumption of sugar-sweetened beverages (SSBs) increases the risk of obesity in children and adults alike [6], and is one of the main drivers of the worldwide epidemic of obesity and diabetes [7, 8]. Affordability is a decisive factor in differences among countries in terms of per capita SSB buying and consumption habits and prevalence of obesity [9]. A total of 19% of Spaniards are regular consumers of SSBs, but this figure rises to almost 40% among the child and adolescent populations [10], something that makes these beverages one of the main sources of energy in these groups [11]. SSBs provide empty liquid calories, with negligible satiating power, altering the body’s regulatory capacity to maintain the necessary balance between energy intake and expenditure [12]. Long-term consumption of SSBs also increases the risk of cardiovascular diseases and cancer, with mortality due to these diseases being 15 to 20% higher among daily consumers of such drinks [13]. While Spanish dietary guidelines recommend occasional consumption of SSBs and ultraprocessed foods, the content of added sugars and ultraprocessed foods in household purchases almost tripled during the last decades [14].

In its report entitled *Fiscal Policies for Diet and Prevention of Noncommunicable Diseases*, the World Health Organisation (WHO) urges countries to levy specific excise taxes on SSBs [15]. To the extent that this type of tax is levied at a rate calculated according to product volume (volumetric taxes) rather than retail price, it discourages SSBs being replaced by other similar, but cheaper, substitutes, such as white-label or own-brand drinks. The WHO also advises that the increase in price be at least 20%, a threshold above which reductions in the consumption of SSBs tend to be more than proportional and there is an appreciable fall in energy intake. In March 2017, the Autonomous Region (*comunidad autónoma*) of Catalonia approved the introduction of a tiered excise tax on SSBs for public health reasons. The rate at which the tax is levied varies according to sugar content, so that the tax is payable by consumers at a statutory rate of 8 cents per litre for beverages having a sugar content of 5 to 8 g per 100 mL, rising to 12 cents per litre for beverages having a sugar content in excess of 8 g [16]. Whereas fruit drinks, sports drinks, tea and coffee, energy and vegetable drinks, sugar-sweetened milk drinks (with or without fruit juice), shakes, flat and carbonated soft drinks, and flavoured water are all subject to the tax, natural fruit juices, fermented milk drinks and drinking yoghurts are exempt.

Currently, more than 50 countries across five continents have introduced some type of tax on SSBs, mostly using a tiered design as in Catalonia [17]. Recent systematic reviews of real-world studies (natural experiments, intervention studies and prospective cohort studies) have concluded that SSB taxes are effective in reducing SSB purchases and consumption at short and medium term, from months to around 2 years after their implementation, suggesting a greater effect for volumetric tiered taxes [18–20]. Two studies assessed the impact of the Catalonian tax, based on purchase data sourced from similar supermarket chains having a market share of around 10% in Catalonia, at 3 months and at 1 year of the tax’s introduction, and observed overall declines of 7.7% in the purchase of SSBs and 2.2% in sugar purchases from beverages, with a partial substitution effect attributable to sugar-free drinks (zero/lights) [21, 22]. Another study, conducted on samples from two low-income districts of Madrid and Barcelona, reported an annual reduction of 39% in the frequency of regular consumers of SSBs [23]. In the first year of the tax’s introduction, these studies observed a moderate price increase of 2 to 17.5%, depending on the type of drink and size of receptacle, showing that the tax has not always been fully passed on to the consumer. Two years after of the tax’s introduction, another study, using data drawn from the Ministry of Agriculture food-consumption panel, reported a decrease of 12.1% in the purchase of sugary cola drinks and an increase of 17% in the purchase of light cola drinks [24].

The aim of this study was thus to evaluate the long-term effect of the Catalonian excise tax on the price and purchase of SSBs and their possible substitutes. To this end, we extended the reach of previous research of data sourced from the Ministry of Agriculture food-consumption panel on a random sample of 12,500 households across Spain, limited to cola drinks, to include fruit drinks and water across the period January 2013–November 2020, three and a half years after the tax implementation.

## Methods

### Study design and sample

The designated aim of the Ministry of Agriculture food-consumption panel is to ascertain the direct demand for food by Spanish households. “Household” is defined as any person or group of people who jointly occupy a family dwelling, wholly or in part, and consume food and other goods funded by a common budget. The sample was made up of 12,500 households that recorded their daily purchases with optical barcode readers [25]. All sample households must have been enlisted and been active participants in the panel at least for 9 months. The sampling procedures were aiming to obtain a sample representative of the national households in terms of socio-economic level, number of members, age and activity of the person responsible for food purchases, and presence and age of children. The sample was randomly selected in two stages: in the first, the panel team selected the survey points (towns and villages in which there were one or more collaborating households) according to the size of the populations (from less than 2000 to more than 500,000 inhabitants) for each of the autonomous regions of Spain; and in the second, they selected participants at each of the designated points. The territorial units were defined in accordance with the European Regulation on the establishment of a common classification of territorial units for statistics (Official Journal of the European Union - *Diario Oficial de la Unión Europea/*DOUE 2013). A sample distribution was required that would allow for parameters to be calculated at the level of Spain’s 17 autonomous regions, by type of habitat (number and composition of the household members as well as the age of the person responsible for food purchases). Consequently, the sample was structured proportionally to population strata, as defined by the following socio-demographic variables: autonomous region; size of the survey points’ populations; socio-economic level of the household; number of household members; age and activity of the person responsible for making purchases; and presence and age of children (see Additional file). To ensure that the existing diversity in Spain was represented, the sample was given a wide territorial spread in proportion to the population that inhabited the different geographical areas, yielding a total of 2241 survey points across the country. The food panel provides aggregated monthly data data broken down by autonomous region, which is publicly available at the web page of the Ministry of Agriculture from January 2013 to November 2020 (https://www.mapa.gob.es/app/consumo-en-hogares/consulta11.asp), allowing for a controlled interrupted temporal series study design [26].

### Data-collection and study variables

Using optical barcode readers, the participating households collected data on the following variables for each item of food or drink acquired in the homes: product; amount purchased; expense incurred; and type of establishment at which the purchase was made. For each drink acquired, the food-consumption panel publishes aggregated monthly data broken down by autonomous region for the following derived variables: total product volume; mean product price per litre; penetration (percentage of purchasing households); and per capita consumption (amount purchased). Our analysis included fruit drinks (drinks with a variable, usually very small, content of juice) and cola drinks, due to the fact that these were the only beverages for which data was supplied with a breakdown by sugar content (i.e., with sugar vs. sugar-free or light), and bottled water. The study period covered the interval from January 2013, 5 years and 4 months before the tax’s introduction, to November 2020, three and a half years after its introduction. To assess the effect of the tax on sugary drinks and their possible substitutes, we created three composite variables, namely, SSBs for taxed beverages, by adding purchases of sugary cola and fruit drinks, non-sugar-sweetened-beverages (NSSBs), by adding purchases of free-sugar or light cola and fruit drinks, and bottled water.

### Statistical analysis

Monthly per capita purchases of bottled water, SSBs and NSSBs were calculated by totalling the amount purchased for all beverages included in each category. The mean price per litre was also calculated for each category. Descriptive annual statistics were then computed for the price and per capita purchases of the beverages included in the study. As there were no risk of contamination and no particular region that could be considered more suitable as control, we applied the synthetic control method to infer the causal impact of the intervention, based on a Bayesian structural time-series model which predicts the counterfactual response that would have occurred had no intervention taken place [27]. Using this method, sixteen time series, one for each of Spain’s autonomous regions unaffected by the intervention, were weighted according to their fit to the outcome of interest in the period before the tax’s introduction, and then combined into a composite time series. We then computed the posterior distribution of the predicted (counterfactual) time series, given the value of the Catalonian and control series in the pre-tax period, along with the values of the controls in the post-tax period, which produced a counterfactual estimation of what would have happened in Catalonia in the post-tax period, had the intervention not occurred, thereby effectively adjusting for unmeasured bias and confounding. In this way, we could minimize the risk of selection bias as well as control for secular trends and history bias due to other events affecting all the regions, like the Covid-pandemic [28]. Subtracting the predicted from the observed response during the post-intervention period yielded a semiparametric Bayesian posterior distribution for the causal effect. The absolute and relative differences between the actual observed data and the prediction during the post-tax period were reported as the estimates of the effect, together with their 95% confidence intervals (α level = 0.05). We performed repeated analyses for periods of one, two, three, and three and a half years after the date of the tax’s introduction (May 2017). To test the robustness of the results, we run the model on pre-tax periods, using May 1, 2016 as the date for placebo intervention. We conducted an additional robustness analysis comparing SSBs trends with water trends in Catalonia. All statistical analyses were performed using the CausalImpact computer software package (version 1.2.1) for R, version 4.0.1.

## Results

SSBs accounted for 23.2% of the total purchase volume of non-alcoholic drinks in Spain before the tax introduction, and 19.6% afterwards; while figures for NSSBs were 22.9, and 26.2% respectively. Among the beverages analysed, the type most purchased in Spain before the tax was implemented were sugary cola drinks with caffeine, which accounted for 19% of the total purchase volume of non-alcoholic drinks, a figure that fell to 15.9% after the tax’s introduction, being surpassed in this period by light cola drinks with caffeine, with 16.1% of the purchase volume. In Catalonia, the SSBs analysed went from 27.9% of the total purchase volume before the tax to 21.6% after the tax, a relative reduction of 22.6%, whereas in the rest of Spain they went from 22.4 to 19.4%, a reduction of 13.4%. In contrast, the NSSBs analysed in Catalonia went from 20% of the total purchase volume before the tax to 25% after the tax, an increase of 25%, whereas in the rest of Spain these went from 23.4 to 26.3%, an increase of 12.4%. Table 1 shows the monthly averages in annual periods as from the date of the tax’s introduction. The mean price of SSBs went from 0.95 in 2013/2014 to 1.17 €/l. in 2019/2020 in Catalonia, and from 0.83 to 0.95 €/l. in the rest of Spain, a relative increase of 23.2 and 14.5% respectively. The equivalent increase in the price of NSSBs was 15.1 and 15.4% respectively, whereas the price of bottled water hardly varied across the study period. Mean monthly per capita consumption of the SSBs analysed went from 1.86 l. in 2013/2014 to 1.11 l. in 2019/2020 in Catalonia, and from 1.51 to 1.09 l. in the rest of Spain, a relative decline of 40.3 and 27.8% respectively. In contrast, the mean monthly per capita consumption of NSSBs went from 0.99 l. in 2013/2014 to 1.13 l. in 2019/2020 in Catalonia, and from 1.06 to 1.13 l. in the rest of Spain, a relative increase of 14.1 and 6.6% respectively. The mean monthly per capita consumption of bottled water rose by 18.7% in Catalonia and 24% in the rest of Spain.

**Table 1:** Price and monthly consumption of beverages in Catalonia and Spain 2013–2020

Variable	Geographical area	Pre-tax period				Post-tax period		
		2013/14	2014 /15	2015 /16	2016 /17	2017 /18	2018 /19	2019 /20
Price (€/L)								
Sugar-sweetened beverages	Catalonia	0.95	0.99	1.03	1.07	1.09	1.11	1.17
	Rest of Spain	0.83	0.84	0.87	0.89	0.89	0.92	0.95
Sugar-free beverages	Catalonia	0.86	0.90	0.91	0.96	0.94	0.94	0.99
	Rest of Spain	0.78	0.77	0.80	0.83	0.84	0.87	0.90
Water	Catalonia	0.21	0.21	0.23	0.23	0.23	0.23	0.22
	Rest of Spain	0.21	0.20	0.21	0.21	0.21	0.21	0.21
Per capita consumption (L/month)								
Sugar-sweetened beverages	Catalonia	1.86	1.73	1.53	1.31	1.24	1.13	1.11
	Rest of Spain	1.51	1.45	1.36	1.30	1.20	1.09	1.09
Sugar-free beverages	Catalonia	0.99	0.86	0.87	0.76	0.92	1.04	1.13
	Rest of Spain	1.06	1.04	1.07	1.03	1.04	1.06	1.13
Water	Catalonia	6.26	6.42	6.69	6.48	7.34	7.10	7.43
	Rest of Spain	4.17	4.01	4.71	4.93	5.09	5.06	5.17

**Fig. 1:**
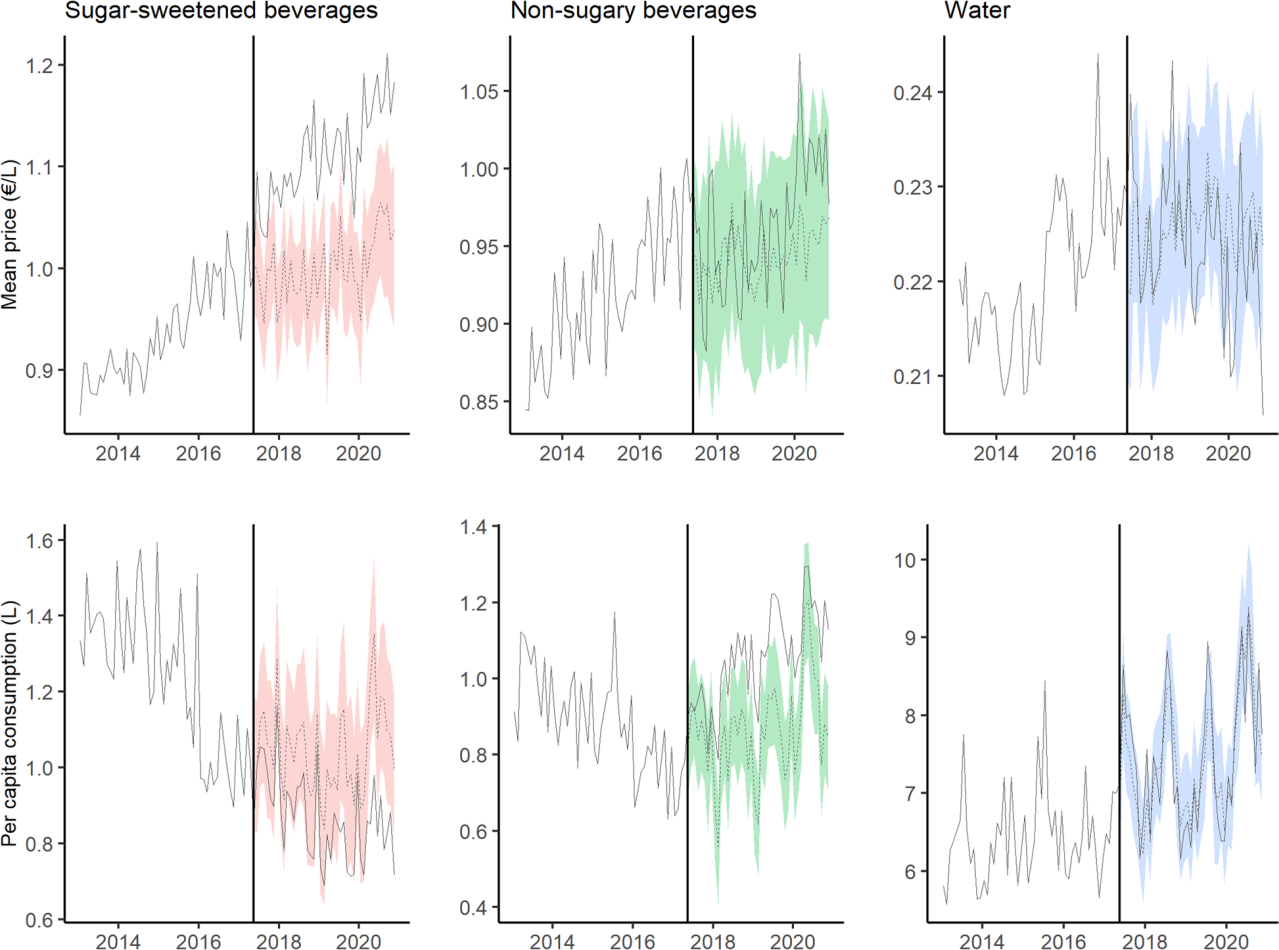
Monthly mean price and per capita purchases of selected sugary drinks, non-sugary drinks and bottled water in Catalonia, Spain: pre- and post-tax periods, and counterfactual predictions. Vertical line: SSB tax start date (May 2017). Continuous line: observed data. Dashed line: counterfactual prediction (mean). Shaded area: counterfactual prediction (95% confidence intervals)

**Fig. 2:**
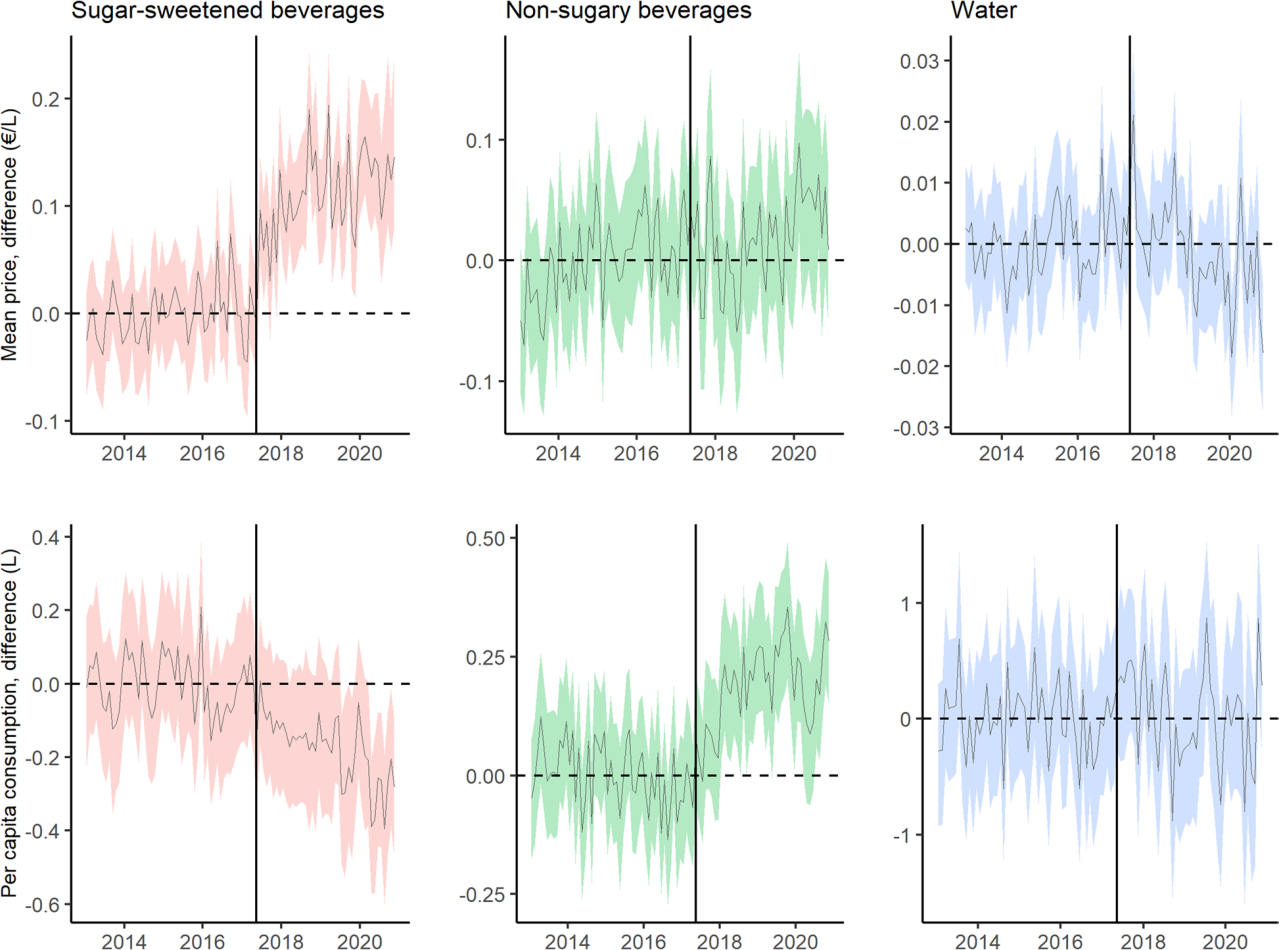
Monthly difference between observed price and per capita consumption of selected sugary drinks, non-sugary drinks and bottled water in Catalonia, Spain, and counterfactual predictions. Vertical line: SSB tax start date (May 2017). Dashed horizontal line: no difference. Continuous line: difference between observed values and counterfactual prediction. Shaded area: 95% confidence intervals of the difference

**Fig. 3:**
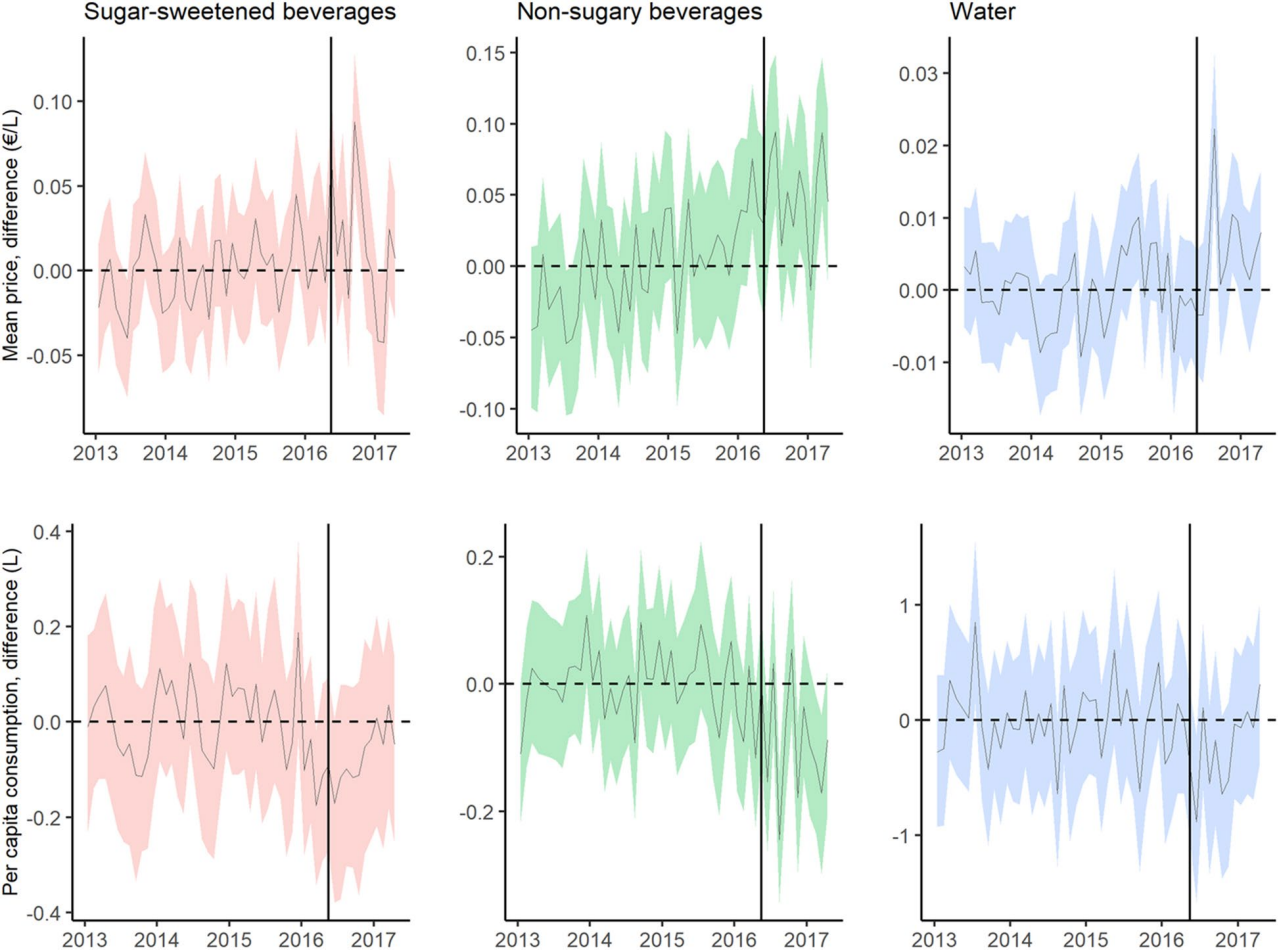
Placebo test by date of intervention (May 1, 2016). Monthly difference between observed price and per capita consumption of selected sugary drinks, non-sugary drinks and bottled water in Catalonia, Spain, and counterfactual predictions. Vertical line: SSB tax start date (May 2016). Dashed horizontal line: no difference. Continuous line: difference between observed values and counterfactual prediction. Shaded area: 95% confidence intervals of the difference

**Fig. 4:**
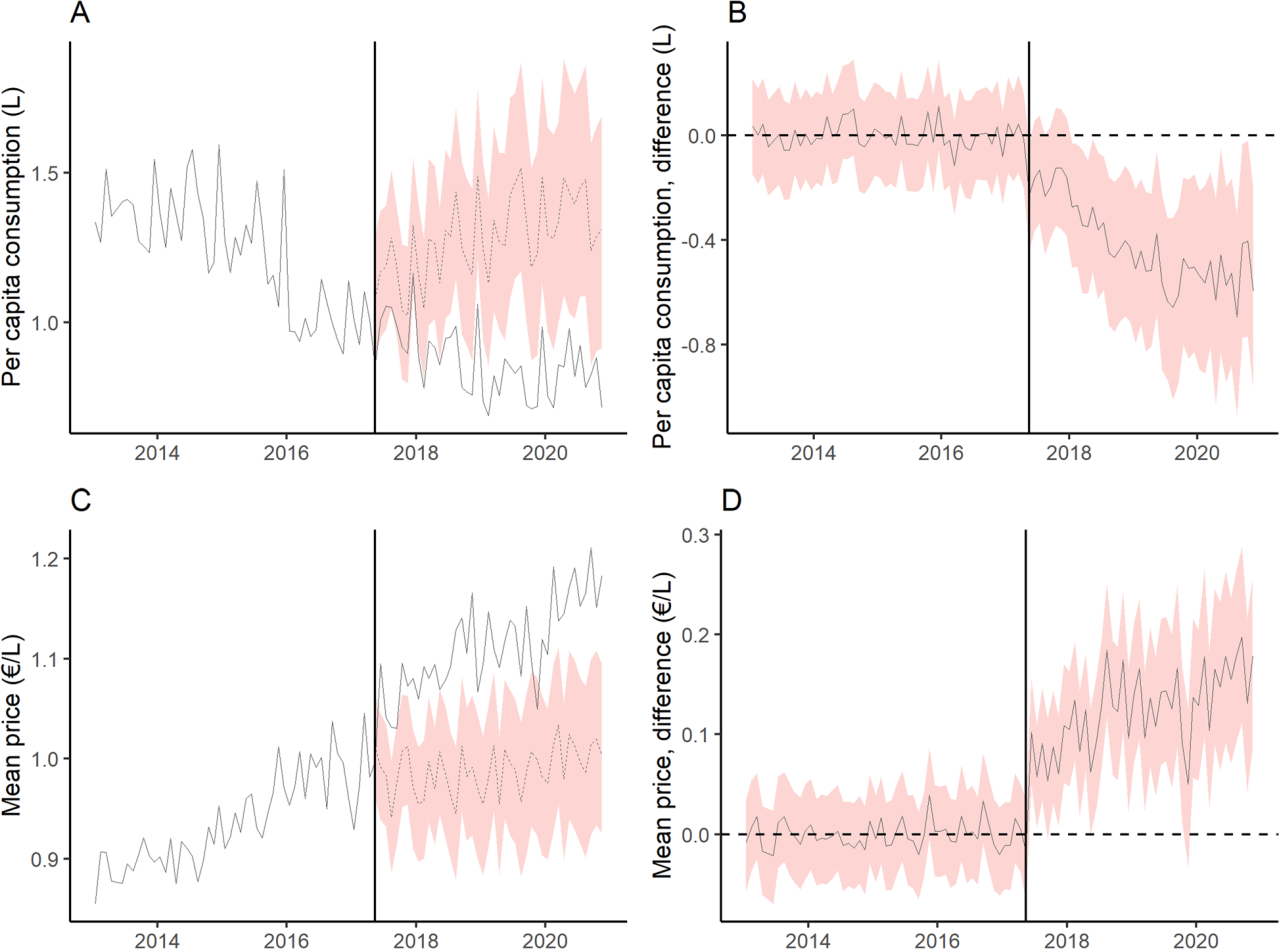
Observed trends and counterfactual predictions of Sugar-Sweetened Beverages (SSB) consumption and price, using water as the control group, Catalonia. **A and C**: observed trends and counterfactual predictions of SSB consumption (**A**) and price (**C**), using water as the control group. Continuous line: observed data. Dashed line: counterfactual prediction (mean). Shaded area: counterfactual prediction (95% confidence intervals). Vertical line: SSB tax start date (May 2017). **B and D**: difference between observed trends and counterfactual predictions of SSB consumption (**B**) and price (**D**), using water as the control group. Dashed horizontal line: no difference. Continuous line: difference between observed values and counterfactual prediction. Shaded area: 95% confidence intervals of the difference. Vertical line: SSB tax start date (May 2017)

Figure 1 shows the monthly mean price and per capita purchases of SSBs, NSSBs and bottled water in Catalonia (Spain), during the pre- and post-tax periods, and the counterfactual predictions. The price of SSBs increased and their consumption decreased progressively over time, while the opposite trends were observed for NSSBs. Figure 2 shows the difference in absolute terms between the observed and counterfactual price and purchases. Compared to the predicted figures, per capita purchases of SSBs fell by 0.17 l (95% CI: − 0.24, − 0.09) three and a half years after implementation of the SSBs excise tax in Catalonia, a relative decline of 16.71% (95% CI: − 23.18, − 8.74). Mean SSBs prices rose by 0.11 €/L (95% CI: 0.09, 0.14), a relative increase of 11.03% (95% CI: 9.02, 14.05). While there were no changes in mean NSSBs prices, NSSBs consumption grew by 0.19 l per capita (95% CI: 0.16, 0.22), a 21.73% increase (95% CI: 18.25, 25.54). No variations in bottled water price or consumption were detected. The effects on price and purchases of SSBs, and on purchases of NSSBs were progressively greater over time, with SSB purchases decreasing by 10.37% at 1 year, 12.31% at 2 years, 15.26% at 3 years, and 16.71% at three and a half years of the tax’s introduction (Table 2). Figure 3 shows the results of the test of robustness, using May 1, 2016 as the date for placebo intervention. While there were no changes in mean consumption of SSBs and water, an increase in NSSBs mean prices was observed with a parallel decrease in NSSBs mean consumption. Figure 4 shows the results of the second test of robustness, using water as the control group. An increase in SSBs mean price was detected with a decrease in SSBs mean consumption progressively greater over time.

**Table 2:** Absolute and relative effect of the 2017 Catalonian SSB tax on price and purchases of sugar-sweetened beverages, non-sugar-sweetened beverages and bottled water after 1, 2, 3 and 3.5 years

Category	Variable	Years	Observed	Predicted (95% CI)	Absolute effect (95% CI)	Relative effect (95% CI)	*p*
SSBs	Mean price (€/L)	1	1.06	0.99 (0.96 to 1.01)	0.08 (0.06 to 0.10)	7.82% (5.78 to 10.14%)	< 0.01
		2	1.09	0.99 (0.97 to 1.01)	0.10 (0.08 to 0.12)	10.12% (8.27 to 12.38%)	< 0.01
		3	1.1	0.99 (0.97 to 1.01)	0.11 (0.09 to 0.13)	10.87% (9.08 to 13.08%)	< 0.01
		3.5	1.11	1.00 (0.97 to 1.02)	0.11 (0.09 to 0.14)	11.03% (9.02 to 14.05%)	< 0.01
	Per capita consumption (L)	1	0.95	1.06 (0.99 to 1.13)	−0.11 (−0.18 to −0.04)	−10.37% (−17.07% to −3.81%)	< 0.01
		2	0.9	1.02 (0.94 to 1.10)	−0.13 (− 0.20 to − 0.04)	−12.31% (− 19.63% to −4.30%)	< 0.01
		3	0.87	1.03 (0.95 to 1.10)	− 0.16 (− 0.23 to − 0.07)	−15.26% (−22.44% to −7.10%)	< 0.01
		3.5	0.87	1.04 (0.96 to 1.11)	− 0.17 (− 0.24 to − 0.09)	− 16.71% (−23.18% to −8.74%)	< 0.01
NSSBs	Mean price (€/L)	1	0.95	0.94 (0.91 to 0.98)	0.01 (− 0.03 to 0.03)	0.64% (− 3.33 to 3.45%)	0.31
		2	0.94	0.94 (0.92 to 0.97)	0.00 (− 0.03 to 0.03)	0.48% (−2.69 to 2.81%)	0.31
		3	0.96	0.94 (0.92 to 0.97)	0.01 (− 0.02 to 0.04)	1.49% (−1.97 to 3.75%)	0.17
		3.5	0.96	0.94 (0.92 to 0.98)	0.02 (− 0.02 to 0.04)	1.89% (−1.84 to 4.40%)	0.14
	Per capita consumption (L)	1	0.92	0.81 (0.76 to 0.85)	0.11 (0.07 to 0.16)	14.06% (8.97 to 20.18%)	< 0.01
		2	0.98	0.82 (0.78 to 0.86)	0.16 (0.12 to 0.20)	19.37% (14.94 to 24.66%)	< 0.01
		3	1.03	0.85 (0.82 to 0.89)	0.18 (0.15 to 0.22)	21.24% (17.26 to 25.25%)	< 0.01
		3.5	1.05	0.86 (0.83 to 0.89)	0.19 (0.16 to 0.22)	21.73% (18.25 to 25.54%)	< 0.01
Water	Mean price (€/L)	1	0.23	0.22 (0.22 to 0.23)	0.00 (0.00 to 0.01)	1.56% (0.10 to 2.98%)	0.02
		2	0.23	0.22 (0.22 to 0.23)	0.00 (0.00 to 0.00)	0.64% (−0.55 to 1.94%)	0.16
		3	0.22	0.23 (0.22 to 0.23)	0.00 (0.00 to 0.00)	−0.20% (−1.50 to 1.42%)	0.37
		3.5	0.22	0.23 (0.22 to 0.23)	0.00 (0.00 to 0.00)	−0.66% (− 1.98 to 0.95%)	0.18
	Per capita consumption (L)	1	7.3	7.09 (6.86 to 7.33)	0.21 (−0.03 to 0.44)	3.01% (− 0.44 to 6.14%)	0.05
		2	7.22	7.16 (6.89 to 7.44)	0.05 (−0.22 to 0.33)	0.77% (−3.09 to 4.65%)	0.36
		3	7.34	7.27 (6.96 to 7.59)	0.07 (−0.26 to 0.38)	0.92% (− 3.51 to 5.23%)	0.34
		3.5	7.47	7.42 (7.11 to 7.73)	0.04 (−0.26 to 0.35)	0.59% (−3.56 to 4.78%)	0.40

*SSBs* Sugar sweetened beverages, *NSSBs* Non-sugar-sweetened beverages, *Years* Years after introduction of the 2017 SSB tax

## Discussion

Three and a half years after the introduction of the Catalonian excise tax on SSBs, there was a mean increase of 11% in the price of taxed beverages, without any change in the price of untaxed beverages, in comparison with the trend observed in the remaining Spanish regions, where the tax was not applied. Per capita consumption of taxed beverages fell by 16.7%, while that of NSSBs rose by 21.7%. None of the parameters analysed registered any change for bottled water.

Our data show that the effect of the tax on the reduction in purchases became progressively greater across the three and a half years. The fall of 10.4% 1 year after implementing the tax was slightly greater than that of 7.7% observed by Vall et al. at 3 months [22], but the difference grew until doubling at the 3-year mark. As was to be expected, the fall of 12.3% in purchases of SSBs 2 years after the tax’s introduction was very similar to that reported by Puig-Codina et al. for sugar cola drinks [24]. Rational models of addictions show that the impact of price rises on consumption is greater in the long term than in the short term, a phenomenon that has been substantiated for both illegal drugs such as cocaine and for legal drugs such as tobacco [29, 30]. Our results confirm that this is also the case for sugar-sweetened beverages, in line with what has previously been observed in Mexico, where the reduction in purchases of SSBs went from 5.5% at 1 year of the tax’s introduction to 9.7% at 2 years [31], and in California, where frequency of daily consumption declined by 0.30 times per day after 1 year and 0.56 times per day after 3 years [32]. This parallelism may be due to the addictive properties of sugar: it interferes with the normal functioning of the leptin and ghrelin hormones, which regulate hunger and satiety, and induces a response similar to that of other drugs in the brain’s dopaminergic reward system, stimulating the individual to consume increasingly more [33]. The COVID-19 pandemic brought about no change in the trend of the tax’s effect, whose impact continued to grow during and after confinement, a period during which there was a slight improvement in adherence to the Mediterranean diet by the Spanish population [34], though without modifications in the consumption of SSBs among adolescents, the main market for these drinks [35]. A SSBs tax has been proposed, among other food policies, to prevent obesity and the main nontransmissible diseases in Spain [36]. The fell in SSBs consumption with the Catalonian tax, if extended to the rest of autonomous regions of the country, could help to curb the epidemic of obesity in Spain.

In two of the studies that analysed the effect of the Catalonian tax, prices increased by around 7.5% for small receptacles (less than 1 L) and 17.5% for large receptacles [22, 23]. In our study, which did not allow for distinction by size of receptacle but instead collected data on a broad-based, nationally representative sample of households that bought their products at all types of sales outlets, the mean increase in price was 7.82% after the first year and 11.03% after three and a half years, figures within the range of previous studies. The effect on per capita purchases of SSBs observed by us is perfectly compatible with the price elasticity of demand of − 1.21 reported by a recent review on the topic, which predicts a 12% reduction in demand with a 10% increase in price [37]. Furthermore, in the case of time-series studies, a systematic review of natural experiments with SSB taxes observed a mean decline of 10.7% in purchases of SSBs in response to a 10% increase in the price, and a 14.0% decline in response to taxes with a sugar-concentration threshold [20]. Although the fall in purchases was higher than these figures in our study, the effect of the tax might be overestimated, bearing in mind that consumption of beverages in small receptacles, where the percentage increase in the price is far lower, tends to be underestimated, since the panel does not cover consumption outside the home, whether at vending machines or in bars, cafés, restaurants and other leisure venues. Although we have no data on the impact of these changes on real consumption and their effects on obesity, modelling studies in the USA and UK have reported that a 20% tax on SSBs would lead to 2.6 and 2.7% decrease in national obesity levels respectively [37, 38].

Our data highlight an important NSSB substitution effect, purchases of which increased by more than 20%, in line with the findings of Puig-Codina et al. for light cola drinks [24]. In the study by Vall et al., around 21% of the reduction in SSB purchases was replaced by an increase in sales of NSSBs [22]; and while there was no increase in the frequency of regular NSSB consumers in low-income districts of Barcelona, 6% of those who reduced their consumption of SSBs reported that they had replaced these with untaxed beverages [23]. The effects of substitution vary widely among countries and the types of beverage considered [20], with increases in consumption that can range from 2% in Mexico to 5.2% in Barbados and 29% in California [31, 32, 39]. These variations may be due to differences in the type of tax (whether or not it affects NSSBs), the methodology of the studies (type of beverage studied and data-collection method) and the socio-economic or cultural characteristics of the countries. Although NSSBs provide no calories to the body, systematic reviews do not clearly support the potential benefits of non-nutritive sweeteners for weight management and show that consumption of artificially-sweetened beverages is associated with a higher risk of hypertension, diabetes, obesity and metabolic syndrome [7, 40–42]. In the Women’s Health Initiative cohort of women, a higher intake of artificially-sweetened beverages was associated with increased risk of stroke, coronary heart disease and all-cause mortality [43]. Hence, the potential health benefits of the tax could be counteracted, totally or in part, by increased consumption of artificially-sweetened beverages. In Philadelphia, with a tax on sugar-sweetened and artificially-sweetened beverages, there was a fall in the sales of both types of beverages [44].

### Strengths and limitations

The strengths of this study consist of the use of a large-sized sample representative of the Spanish population, with monthly data series available from January 2013, 5 years and 4 months before the tax’s introduction, on SSBs and their possible substitutes, such as NSSBs and bottled water. Furthermore, the use of a synthetic control group to estimate the expected trend in Catalonia from May 2017, on the assumption that the tax had not been implemented, based on the series for the remaining 16 autonomous regions in Spain, before and after the tax, made it possible to control for the effect observed due to local trends, seasonality, and time-varying confounding factors [27]. Furthermore, a placebo test was conducted on pre-tax periods and a second one using water as the control group. Even so, our study has some limitations. Firstly, only beverages with data broken down by sugar content (i.e., with sugar vs. sugar-free) could be analysed. That said, however, the beverages included are the most representative of each category and, as a whole, account for 47% of all purchases of non-alcoholic drinks. Secondly, our study did not include consumption at establishments/points of sale not intended for home purchases, such as hospitality venues or drink vending machines. Thirdly, we had no data on potential effect modifiers, such as the composition and socio-economic status of the households concerned. Fourth and lastly, we were unable to analyse the possible effects of replacement by cheaper brands, larger-sized receptacles or beverages with a lower sugar content, since there were no data available on these variables.

## Conclusions

The excise tax on SSBs in Catalonia translated as a moderate increase in their price (11%), in comparison with price trends in the rest of Spain, and as a progressive impact on purchases of SSBs, which fell by as much as 16.7% at three and a half years of the tax’s introduction. Similarly, there was an NSSB substitution effect, with the purchases of these drinks increasing by 21.7%. Our results corroborate the effectiveness of an SSB tax observed in previous studies, and show that it has a sustained and progressive impact over time. The observed NSSB substitution effect should be borne in mind when it comes to considering application of this type of tax to the rest of Spain, since beverages with artificial sweeteners are not exempt from health risks.

## Supplementary Information

**Additional file 1.** Characteristics of households in the Spanish population, 2020.

## Data Availability

The datasets generated and/or analysed during the current study are available from the corresponding author on reasonable request.

## Abbreviations

COVID-19: coronavirus disease 2019
NSSBs: Non-sugar-sweetened beverage
SSBs: Sugar-sweetened beverage
WHO: World Health Organization
