# Supplementary material for: Effect of excise tax on sugar-sweetened beverages in Catalonia, Spain, three and a half years after its introduction
Source: Int J Behav Nutr Phys Act. 2022 Mar 12;19:24. doi: 10.1186/s12966-022-01262-8 (PMC8917362; doi:10.1186/s12966-022-01262-8)
Supplement: Supplementary file 1 — Additional file 1. Characteristics of households in the Spanish population, 2020. [file 12966_2022_1262_MOESM1_ESM.docx]

## Information regarding the sample

The designated aim of the Ministry of Agriculture food-consumption panel is to ascertain the direct demand for food by Spanish households. “Household” is defined as any person or group of people who jointly occupy a family dwelling, wholly or in part, and consume food and other goods funded by a common budget. The sample was made up of 12,500 households that recorded their daily purchases with optical barcode readers. All sample households must have been enlisted and been active participants in the panel at least for 9 months.

The sampling procedures were aiming to obtain a sample representative of the national households in terms of socio-economic level, number of members, age and activity of the person responsible for food purchases, and presence and age of children (see below characteristics of households in the Spanish population and the study sample in 2013, at the beginning of the study).

The sample was randomly selected in two stages: in the first, the panel team selected the survey points (towns and villages in which there were one or more collaborating households) according to the size of the populations (from less than 2,000 to more than 500,000 inhabitants) for each of the autonomous regions of Spain; and in the second, they selected participants at each of the designated points. The territorial units were defined in accordance with the European Regulation on the establishment of a common classification of territorial units for statistics (Official Journal of the European Union - *Diario Oficial de la Unión Europea/*DOUE 2013). A sample distribution was required that would allow for parameters to be calculated at the level of Spain’s 17 autonomous regions, by type of habitat (number and composition of the household members as well as the age of the person responsible for food purchases). Consequently, the sample was structured proportionally to population strata, as defined by the following socio-demographic variables: autonomous region; size of the survey points’ populations; socio-economic level of the household; number of household members; age and activity of the person responsible for making purchases; and presence and age of children. To ensure that the existing diversity in Spain was represented, the sample was given a wide territorial spread in proportion to the population that inhabited the different geographical areas, yielding a total of 2241 survey points across the country. The food panel provides aggregated monthly data data broken down by autonomous region, which is publicly available at the web page of the Ministry of Agriculture from January 2013 to November 2020 (<https://www.mapa.gob.es/app/consumo-en-hogares/consulta11.asp>).

| **Characteristics of households in the Spanish population, 2020.** | |  |
| --- | --- | --- |
|  |  |  |
| **Autonomous Communities** | **% Households** | **Households** |
| **CATALUÑA** | 16% | 3.033.859 |
| **ARAGON** | 3% | 542.015 |
| **BALEARES** | 2% | 461.625 |
| **VALENCIA** | 11% | 2.023.707 |
| **MURCIA** | 3% | 550.667 |
| **ANDALUCIA** | 17% | 3.259.221 |
| **MADRID** | 14% | 2.632.366 |
| **CASTILLA LA MANCHA** | 4% | 787.033 |
| **EXTREMADURA** | 2% | 431.860 |
| **CASTILLA LEON** | 5% | 1.016.132 |
| **GALICIA** | 6% | 1.091.575 |
| **ASTURIAS** | 2% | 453.725 |
| **CANTABRIA** | 1% | 242.540 |
| **PAIS VASCO** | 5% | 909.780 |
| **LA RIOJA** | 1% | 130.114 |
| **NAVARRA** | 1% | 258.556 |
| **CANARIAS** | 5% | 857.895 |
| **TOTAL** | 100% | 18.682.670 |
|  |  |  |
| **Size of habitat** |  |  |
| **< 2000 HABIT.** | 6% | 1.169.567 |
| **2000 A 10000** | 15% | 2.717.996 |
| **10001 A 100000** | 38% | 7.089.528 |
| **100001 A 500000** | 24% | 4.450.545 |
| **+ DE 500000** | 17% | 3.255.034 |
| **TOTAL** | 100% | 18.682.670 |
|  |  |  |
| **Socioeconomic status** |  |  |
| **High and upper-middle** | 16% | 3.047.143 |
| **Middle** | 31% | 5.735.580 |
| **Lower-middle** | 27% | 5.005.087 |
| **Low** | 26% | 4.894.860 |
| **TOTAL** | 100% | 18.682.670 |
|  |  |  |
| **Number of members** |  |  |
| **1 member** | 26% | 4.871.093 |
| **2 members** | 31% | 5.710.512 |
| **3 members** | 20% | 3.819.107 |
| **4 members** | 17% | 3.191.901 |
| **5 or more members** | 6% | 1.090.057 |
| **TOTAL** | 100% | 18.682.670 |
|  |  |  |
| **Age of the person responsible for making purchases** |  |  |
| **< 35 years old** | 11% | 2.071.189 |
| **35 to 49 years old** | 31% | 5.829.304 |
| **> 50 years old** | 58% | 10.782.177 |
| **50 to 64 years old** | 29% | 5.403.145 |
| **> 64 years old** | 29% | 5.379.032 |
| **TOTAL** | 100% | 18.682.670 |
|  |  |  |
| **Activity of the person responsible for making purchases** |  |  |
| **Employed** | 43% | 8.009.662 |
| **Unemployed or retired** | 57% | 10.673.008 |
| **TOTAL** | 100% | 18.682.670 |
|  |  |  |
| **Presence and age of children** | **%HOGARES** | **HOGARES** |
| **No children** | 74% | 13.885.781 |
| **Children less than 6 years old** | 11% | 2.058.897 |
| **Children from 6 to 15 years old** | 15% | 2.737.991 |
| **TOTAL** | 100% | 18.682.670 |
|  |  |  |

**Characteristics of households in the Spanish population and the study sample, 2013.**

| **Socio-economic level** | Spain households (%) | Sample households (%) |
| --- | --- | --- |
| High | 18,2 | 18,4 |
| Medium | 30,7 | 30,6 |
| Low medium | 25,7 | 25,8 |
| Low | 25,4 | 25,2 |
| **Total** | 100 | 100 |

| **Size of the populations** | Spain households (%) | Sample households (%) |
| --- | --- | --- |
| < 2000 HABIT. | 8,3 | 8 |
| 2000 A 10000 | 16,4 | 15,9 |
| 10001 A 100000 | 34,4 | 35,3 |
| 100001 A 500000 | 22,2 | 22,8 |
| >500000 | 18,8 | 18 |
| **Total** | 100 | 100 |

| **Household composition** | Spain households (%) | Sample households (%) |
| --- | --- | --- |
| Independent young people | 4,3 | 4,3 |
| Young couples without children | 6,8 | 6,6 |
| Couples witht children <7 years old | 16,9 | 17 |
| Couples witht children 7-17 years old | 20,2 | 20,2 |
| Couples witht children >17 years old | 9,9 | 9,9 |
| Single parent households | 5 | 5,4 |
| Adult couples without children | 8,5 | 8,7 |
| Independent adults | 5,9 | 6 |
| Retired | 22,4 | 21,9 |
| **Total** | 100 | 100 |
